# Surgery of the Intestines

**Published:** 1894-06-02

**Authors:** 


### SURGERY OF THE INTESTINES.

**Intussusception.**—Mr. Barker records[1] 7 cases in children that have been under his care. Three were females and 4 males. All were infants under 18 months except one boy of 4 years and another of 12 years of age. One was treated by repeated injection and inflation alone, with apparent reduction each time until gangrene and collapse came on, with speedy death. Six were treated by laparotomy after injection had completely failed. Of these 3 recovered perfectly, one aged 4 years, one 5 months, the last 7 months. In addition to these he has collected 18 cases occurring in children at the University College Hospital, making 25 cases with his own, all under 13 years of age. Of these 13 recovered and 12 died. The treatment adopted was either manipulation (1 case) or injection of air or water (7 cases), followed in case of failure by laparotomy (11 cases). In 5 cases laparotomy was done without any previous attempts at reduction; 2 of these were of the enteric variety; 4 died, 1 recovered. Of the 19 cases where injection and manipulation had been tried, followed by laparotomy where necessary (11 cases), 7 died and 12 recovered. Of these 12 recoveries, 7 followed manipulation or injection alone, 5 followed laparotomy where these had failed. Of the 8 fatal cases, 7 deaths followed laparotomy, 1 followed repeated injection. Taken in the gross, there were therefore 17 laparotomies with 11 deaths, and 8 injections or manipulations alone with 1 death. Of course the majority of the recoveries are credited to the injection method, inasmuch as laparotomy was only resorted to when the latter had failed. From a study of these and other cases Mr. Barker deduces the following conclusions:—

1. That in all cases of intus-susception in children injection of water or manipulation should be at once resorted to if the patient is seen within a few hours of the onset of the strangulation.

2. That if these means fail after a fair trial, not too much prolonged, laparotomy should be at once done as the safest treatment.

3. That there is a certain proportion of cases amongst all the varieties of intus-susception which no amount of injection will relieve, or in which injection would be dangerous, and these can only be dealt with by opening the abdomen.

Mr. Mortimer thinks[2] that the best way of performing injection in these cases is by means of a tube and funnel, the latter not being raised more than three feet above the level of the abdomen. Ludwig Hektoen describes[3] an unique case of ideal invagination, in which there were four symmetrical and equidistant intus-susceptions. All the invaginations were ascending, and had healed by sequestration of the intussusception, and by firm union between the intestinal ends, all reparative and compensatory changes being so uniformly advanced as to lead to the conclusion that the invaginations are of simultaneous origin. It is not possible to determine positively whether they are primarily inflammatory or persistent agonal intussusceptions. The clinical history furnishes many incidents that point to a genesis similar to that of invaginations of the dying, as there may have been periods of impending extinction of life, as well as of arrested or of riotous peristalsis without any immediately threatening symptoms. Mr. Bryant describes[4] two cases of intus-susception of the large intestine, due to the presence of a papillomatous growth, successfully reduced by the introduction of the hand into the rectum after removal of the growth by drawing it down and ligaturing it at its base. Mr. Bryant thinks that in each of these cases the cause of the intus-susception was papillomatous, and expresses the opinion that those growths were not infrequent in the large intestine and rectum, and that in all cases of intus-susception in adults they were to be looked for. He advocated the treatment of reduction by passing the hand into the rectum, but this, he has found, is only possible in females. He had little difficulty in these cases in introducing his hand—which measured when closed for introduction 9¼ inches—into the rectum, and in only one case of about a dozen in which he has made the attempt was there any subsequent troublesome want of control in the anal sphincter. He has never succeeded in passing his hand into the rectum of a male patient. Mr. Creswick Williams succeeded[5] in reducing an intus-susception by generating carbon dioxide within the bowel. For the previous twenty-four hours the child—which was eight months old—had had violent straining, and passed slimy stools tinged with blood. There was a sausage-shaped swelling felt midway between the umbilicus and crest of the ilium, on the left side. Mr. Williams procured two basins, each containing about half a pint of warm water. In one he dissolved a drachm and a half of citric acid, and in the other two drachms of bicarbonate of soda. A flexible catheter was attached to a Higginson's enema syringe and passed into the bowel to the extent of 9 inches, and he then injected first the acid and afterwards the alkaline solution. The generation of carbonic acid gas which ensued caused a perceptible distension of the abdomen. The catheter was quickly withdrawn, and the nates were held firmly together for two or three minutes in order to prevent the escape of the gas. After a few minutes the abdominal swelling above described had entirely disappeared. The child ceased to vomit, and in two days passed a natural motion, recovery being complete.

**Jejunostomy.**—Albert describes[6] a new method which he has recently practised in a case of carcinoma of the pylorus, in which the conditions were unfavourable both to resection of the growth and to gastro-enterostomy. After the abdomen had been opened by a transverse incision on the right side, the portion of jejunum which first presented itself was drawn through the opening. This incision was then closed, along the greater part of its extent by provisional suture. An anastomosis was established at the base of the loop of jejunum, between its proximal and distal portions, in order to permit a direct flow of bile and pancreatic secretion from the upper to the lower part of the intestinal canal. A second incision was then made in the abdominal wall parallel with and about one and a half inches above the first incision. The skin between the two openings having been detached from the subjacent structures, the free portion of the loop of jejunum was drawn under the bridge thus formed, and fixed by sutures to the edges of the upper wound. The lower and original wound was afterwards completely closed. The intestinal loop was so disposed that the part corresponding to the anastomosis lay just within the parietal peritoneum, whilst the main and free portion of the loop followed a straight and subcutaneous course to the outer surface of the abdomen. By this plan the author hoped to prevent any subsequent regurgitation of intestinal contents. The attached fundus of the loop was opened by a Paquelin's cautery on the fourth day. The patient progressed favourably for a time, but the malignant disease extended rapidly, and death occurred from exhaustion at the end of the eighth week. A second case is recorded in which this operation was performed for the relief of extreme prostration and hunger, caused by extensive ulceration of the stomach and œsophagus from the action of caustic, but the patient died from collapse in the course of a few hours.

**Circular Enterorhaphy.**—By Paul's method. Mr. Horrocks and Mr. Paul both relate[7] cases in which the lower part of the bowel was invaginated into the upper. Mr. Horrocks' case was one of excision of intestine for malignant disease, and Mr. Paul's case was an enterotomy for gangrenous bowel in a case of hernia. In each instance it was found to be impossible to invaginate the upper into the lower portion of the intestine owing to the dilatation of the former. Mr. Paul thinks that these cases dispose of an objection which has been urged against his operation, namely, that it would not always be possible to invaginate the enlarged proximal end of the bowel into the distal shrunken end, and that in such cases it would therefore be useless. Hitherto it has been thought unsafe in human beings to practise invagination upwards, although it has been shown to be safe in dogs. There are now, however, two cases in which it has been done in this way, and both have made a rapid and complete recovery, and it may therefore be conjectured that it is practically unimportant which way the bowel is invaginated, though Mr. Paul prefers the downward method when feasible, and can only recommend his operation for the small intestine, as most parts of the large intestine are too fixed to permit of a sufficiently free manipulation of the bowel, and are, therefore, unsuitable for it.

Ramaugé publishes[8] the results of his experiments with an aluminium "enteroplexe" of his own invention, and draws the following conclusions from his work: 1. Enterorhaphy is a tedious and difficult operation, with an enormous mortality. 2. Enteroplexy requires but little time; it can be done by any physician, and produces a perfect cicatrisation. 3. The cicatrix is small, linear, and does not cause a lessening of the intestinal calibre. 4. The "enteroplexe," on account of its weight, its form, and its volume, does not endanger the intestine, and is easily passed. 5. The indications for this operation are numerous, and this instrument is applicable to them all. 6. The experiments on animals give remarkable results. 7. This operation is the desideratum of gastro-intestinal surgery.

R. Baracz,[9] with the view of finding some *substitute for decalcified bone plates*, which take too long to prepare, examined various vegetable substances—apple, carrot, potato, beetroot, &c.—and came to the conclusion that turnip is the best material for the purpose. He performed a series of experimental operations on dogs, including five gastro-enterostomies, and three resections of intestine, with entero-anastomosis. Among the five gastro-enterostomies there were four recoveries; the remaining dog died of peritonitis, caused by the escape of the contents of the stomach into the peritoneal cavity at the time of operation. Among the three cases of resection of the intestine there was one death, due to intestinal occlusion, consequent on the anastomosis being made too far from the sutured end. The author considers that the failures had nothing to do with the turnip plates which he used.

In the case of gastro-enterostomy which proved fatal, he examined the seat of anastomosis two days after operation. The plates were found at the points where they had been applied fixed by sutures around the fistula; the stomach plate was soft like boiled carrot, the intestinal plate was somewhat harder. Two dogs were killed fifteen to twenty days after the gastro-enterostomy, and the plates were found neither at the seat of anastomosis nor in the intestinal canal; the sutures had also disappeared. Neither was any trace of plates found in cases where the dogs were sacrificed at a later period—namely, thirty-eight to fifty days after the operation.

**Gunshot Wounds of the Intestines.**—Dr. Miles reports[10] thirteen cases. In speaking of the difficulty of diagnosis in these cases, he says that the most positive means at our command are the hydrogen gas test and exploratory laparotomy. The inflammable gas test is not always conclusive. Wounds of the intestines may exist and yet escape recognition by this method of diagnosis, and, moreover, the concurrent escape of the intestinal contents with the gas, and extravasation in the peritoneal cavity is an accident which may seriously complicate the case. He therefore thinks that exploratory laparotomy offers the quickest and safest method of positive diagnosis. In any case of gunshot wound of the abdomen, one is safe in assuming that the intestines have been wounded if the missile, delivered at short range, has penetrated the anatomical regions overlying this part of the alimentary canal. Operation should be performed in the median line of the abdomen, whenever practicable, and the intestines passed in review from one end to the other, and special examinations should be made of all organs contiguous to the bullet's track. The peritoneal cavity should be cleansed by hand by aid of a full stream of hot sterilised water poured from a bowl or pitcher. Any amount of clean water or blood remaining does less harm than the irritation caused by sponging the cavity dry. Sponges or other absorbent material are not generally used in cleansing the cavity. Of the thirteen cases reported all but one were wounds of the small bowel, and most of them were wounds of great gravity. Eight cases of the thirteen died, but amongst those that recovered there was one suffering from sixteen wounds of the small intestine, one of fourteen, and another of ten—sufficiently strong arguments to sustain the wisdom of operating in these cases. Brady also reports[11] twenty-eight cases of gunshot wound of the intestines treated by abdominal section with nine recoveries. No stress has been laid upon the existence of peritonitis previous to the operation, though this existed in about one-third of the cases operated upon, owing to the long time which had elapsed between the receipt of the injury and the operation. Of three wounds of the stomach all died. Of three wounds of the colon, all recovered; of thirteen wounds of the small intestine, six recovered. He believes that cleansing of the abdominal cavity is of the utmost importance, but objects to the use of absorbent cotton for sponging, as frequently this material has been found in post-mortem examinations where cotton has been used during the operation. Mr. Jonathan Hutchinson, jun., reports[12] a rare case of

**Acute Intestinal Obstruction** due to multiple hydatid cysts. In abdominal section the author found three hydatid cysts projecting from the peritoneum of the mesentery, and extending in a row from the right of the umbilicus to the recto-vesical pouch, in which lay the largest of the three. They were of cartilaginous hardness and ivory whiteness, the largest being of the size of a goose's egg and apparently impacted in the pelvis. The cyst walls were one-third of an inch in thickness, and contained numerous daughter cysts; they were in part calcareous. The cysts were removed and the patient recovered.

[1] Brit. Med. Journ. Feb. 17, 1894, p. 345. [2] Brit. Med. Journ., Feb 24, 1894, p. 438. [3] International Med. Mag., Dec., 1893. [4] Lancet, Feb. 17, 1894, p. 405. [5] Lancet, Mar. 3, 1894, p. 537. [6] Brit. Med. Journ., Jan. 27, 1894; and Wein. Med. Woch, No. 2, 1894. [7] Brit. Med. Journ., Feb. 3, 1894, p. 235. [8] Amer. Journ. of Med. Science, Mar., 1894, p. 333. [9] Brit. Med. Journ., Feb. 24, 1894, p. 30 of "Epitome." [10] Annals of Surgery, Dec., 1893, p. 623. [11] International Journ. of Surgery, Aug. 1893; and Therap. Gazette, Jan., 1894.

